# *Salsola soda* as selenium biofortification crop under high saline and boron growing conditions

**DOI:** 10.3389/fpls.2022.996502

**Published:** 2022-09-26

**Authors:** Gary S. Bañuelos, Tiziana Centofanti, Maria C. Zambrano, Kaomine Vang, Todd A. Lone

**Affiliations:** ^1^United States Department of Agriculture (USDA), Agricultural Research Service, San Joaquin Valley Agricultural Sciences Center, Parlier, CA, United States; ^2^Department of Environmental Sciences and Policy, Central European University, Vienna, Austria; ^3^Center for Irrigation Technology, California State University Fresno, Fresno, CA, United States; ^4^Department of Agricultural Business, Jordan College of Agricultural Sciences and Technology, California State University Fresno, Fresno, CA, United States

**Keywords:** biofortification, *Salsola soda*, selenium, boron, salinity

## Abstract

In California, there is a shortage of good quality water available for irrigated agriculture due to severe drought. Consequently, saline groundwaters and drainage waters containing natural-occurring selenium (Se) and boron (B) salts are being considered as alternative sources of water for irrigation on salt and B tolerant crops like the edible halophyte-agretti (Salsola soda L.). In this multi-year field study, we evaluated agretti grown as a Se-biofortification crop in typical saline/B-laden soils (*1*0 dS m^−1^ and 12 mg B/L) and irrigated with saline (3–8 dS m^−1^) and low-saline water (<1 d/S m) containing B (3–6 mg B/L) and Se (0.02–0.25 mg Se/L) at different evaporation transpiration (Et_*o*_) rates (100, 75, and 50 %, respectively). During the four-year study, fresh biomass yields ranged from 1 to 3 kg/m^2^ and were generally highest with irrigation at 100 % Et_*o*_ with either saline or low-saline water. Tissue Se concentrations ranged from 2 to 3.2 mg Se / kg DW and 0.4–0.5 mg Se/kg DW with saline and low-saline irrigation, respectively. Selenium speciation in plant tissue showed the following: selenomethionine (SeMet) > selenate (SeO_4_) > methylselenocysteine (MeSeCy_*s*_), irrespective of any treatment (i.e., year of planting, saline or low saline irrigation, rate of water application, direct seeding or transplanted). Agretti did not exhibit any toxicity symptoms as indicated by changes in total phenolic concentrations. Total phenolics ranged from 180 to 257 GAE/L and showed no significant differences among all treatments, although they were generally higher at the lowest water treatment (50% Et_*o*_). In regard to toxic ion accumulation, agretti tolerated excessive sodium (Na) and boron (B) and tissue concentrations ranging from 5.5 to 8.8% Na and 60 to 235 mg B/kg DW, respectively. Results from this multi-year study have identified a unique Se-biofortification strategy for producing Se-enriched agretti using saline, B- and Se-laden soil and irrigating with saline and low-saline water, respectively. Successful production of this crop may promote Se- biofortification strategies in poor quality regions where natural- occurring Se is present in soils and in waters used for irrigation.

## Introduction

Presently, about 3.6 billion people suffer water scarcity each year (Boretti and Rosa, 2019) and the situation is expected to become worse in the next few decades (World Water Assessment Programme (Nations Unies), 2018), especially affecting food production. This problem is even more serious for irrigated agriculture in one of the most productive regions of the USA, the San Joaquin Valley (SJV) in Central California. In Central California, there is a shortage of good quality water available for irrigated agriculture due to severe drought, reductions in water allotments, and growing municipal, urban, and environmental demands. Hence, the identification of other water sources, which include high saline water, is considered as an alternative source to water shortages in arid regions of Central California. The potential to use high saline water for irrigation has been studied on field sites in the westside of the San Joaquin Valley (SJV) in Central California since the 1980’s (Oster and Grattan, 2002; Suyama et al., 2007; Ayars and Soppe, 2014). This part of California is unique because of the existence of natural-occurring selenium (Se) and boron (B) salts in soils of the SJV and consequently in ground water and in drainage waters produced from irrigated agriculture. Their presence complicates water reuse strategies and requires selecting crops that can tolerate both the high levels of salinity and B (Díaz et al., 2013). Importantly, the additional presence of soil Se can be a double-edge sword because Se can be toxic to the biological ecosystem at excessive concentrations (as high as 1000 μg Se/L in surface water, Fordyce, 2013), although it is an essential benefit for human and animal nutrition. Selenium, as an intrinsic component of essential seleno proteins, is required in trace amounts for preserving the optimal health and balanced metabolism of mammals (Rayman, 2020). In humans, selenoproteins have functional roles in antioxidant process, protein stability, transcription of mRNA immune system, and other biochemical functions (Broadley et al., 2006; Labunskyy et al., 2014; Kieliszek, 2019). Hence, it is important for humans to maintain adequate levels of Se in their daily diet.

Consumption of meat and food crops are major sources of Se for the world’s population. If natural-occurring soil Se is absorbed by a food crop, a process called Se biofortification occurs (Bañuelos et al., 2017; El-Ramady et al., 2020). Selenium biofortification can be a strategy to combat the low Se status in many parts of the world by producing food crops that are enriched with Se (Broadley et al., 2006). Generally, Se biofortification occurs when some form of Se is exogenously applied to plant or soil (Jiang et al., 2017; Smoleń et al., 2019; Luo et al., 2021), or when crops are grown in Se-laden soils or irrigated with Se-rich waters (Broadley et al., 2006; Zhu et al., 2009; Bañuelos et al., 2017; Hossain et al., 2021). For example, using Se-laden drainage water as an alternative source of water in drought-stricken California, is also a source of Se for Se biofortification strategies. However, identifying crops that are salt and B tolerant, is a prerequisite for any water reuse strategy implemented in the westside of Central California, because of the abundance of geogenic sources of salts.

In this regard, halophytic plants may be an alternative salt tolerant plant species to consider that can be cultivated on saline soil and utilize saline waters (Galvani, 2007; Flowers and Colmer, 2008; Rozema and Flowers, 2008; Díaz et al., 2013). In the westside of Central California, other salt-tolerant forage species, i.e., atriplex, have been sustainably irrigated with Se-enriched saline water to produce Se-enriched annual forage (Watson, 1990; Watson and O’Leary, 1993). Although many halophytic species have primarily been utilized in animal forage (Norman et al., 2013; Attia-Ismail, 2018; Marinoni et al., 2019), a few halophytic plants have had a place in the diet of people around the world (Watson, 1990; Watson and O’Leary, 1993; Panta et al., 2014). In contrast to utilizing halophytes as forage, the halophytic plant, *Salsola soda*, ‘agretti’, native to saline soil in the coastal regions of the Mediterranean basin, is commonly consumed in many parts of Southern Europe, i.e., Italy (Colla et al., 2006; Minuto et al., 2011). The plant is farmed as a vegetable and in folk medicine, *Salsola* species, was used to treat hypertension, constipation and inflammation (Tundis et al., 2009; Iannuzzi et al., 2020).

In 2014, initial greenhouse work by Centofanti and Bañuelos (2015) evaluated agretti’s ability to tolerate irrigation water with high salinity, B, and Se when grown in saline soils. They showed that *Salsola soda* can grow in saline (EC > 10 dS m^–1^) and B-laden soils (10 mg/L^–1^) and tolerate irrigation with saline and B and Se rich water (EC of 3 dS m^–1^, 4 mg B L^–1^ and 0.1 mg Se L^–1^). Moreover, the plant extracted and accumulated Se and Na. In this regard, concentrations of tissue Se ranged from 3 to 5 mg kg^–1^ DW and concentrations of plant Na were as high as 8% DW under high saline growing conditions. Agretti’s ability to extract selected ions, i.e., Se and Na, under saline conditions suggests that the plant may be a candidate for Se biofortification and may also be useful for biologically managing soluble Se and Na added to soils when using saline waters originating from the westside of the SJV.

Based upon the initial research conducted by Centofanti and Bañuelos (2015) on the impact of salinity and B on the accumulation of Se, the current multi-year field Se biofortification study was established on growing agretti under saline soil and saline irrigation conditions. To the best of our knowledge, there have been no investigations on growing agretti as a Se-biofortified crop under saline irrigated field conditions in the westside of the SJV in California. Therefore, the goal of multi-year field study was to determine if agretti can be considered an alternative Se-biofortification crop for growing under organic-like conditions in saline/B and Se laden soils and irrigated with either saline water or low-saline water. We will identify the impact of saline and low-saline waters applied at different rates on agretti’s growth, Se accumulation (including chemical forms of Se accumulated) when producing a Se-biofortified product under saline growing conditions. Results of this study should provide evidence for promoting agretti as an alternative Se-enriched crop grown with either Se-laden saline drainage water or low-saline water in saline and Se-rich soils of the westside of the SJV or in other similar geological regions containing Se, salinity, and, e.g., Colorado, China.

## Materials and methods

### Field experiment

The saline, B- and Se-laden microplots were established at Red Rock Ranch (RRR), Five Points, CA (36°22′59.73″ N and 120°13′44.94″ W). The soil is classified as an Oxalis silty clay loam (fine montmorillonitic, thermic in Pachic Haploxeral with a well-developed salinity profile). Soil salinity at the field site soil ranged from 7 to 16 dS m^–1^, soluble B from 10 to 18 mg L^–1^, and soluble Se from 0.175 to 0.500 mg L^–1^, respectively. The multi-year study took place in 2016, 2017, 2018, and 2021 (described below). The field sites for plantings in 2016 and in 2017 consisted of 18 raised planting beds (30 m long and 180 cm wide), respectively, while 18 raised planting beds (30 m long and 45 cm wide) were used in planting in 2018. In the 2021 planting, the beds consisted of eighteen 15 m long and 45 cm wide (Supplementary Figure 1). Over the course of this four-year field study, agretti *(Salsoda soda* L*.)* seeds were germinated in seedling trays under greenhouse conditions prior to being transplanted into the field microplots. Greenhouse conditions for growing seeds were generally as follows: day/night temperatures 26/20*^o^*C, 16h photo period, 20-30% relative humidity of ambient air, and an average daily 500-800 μmol photons m^–2^ s^–2^ light intensity. Agretti was transplanted as 3-4 weeks old plantlets in the field sites in 2016, 2017, 2018, and direct-seeded in 2021 (described later). For planting, thirty-day old plants were transplanted 20 cm apart in two rows, which were 50 cm apart from each other in year 2016, 2017 and 2018. In 2021, we also did direct seeding (described below) with 1 cm distance between seeds on 9 beds (15 m long and 45 cm wide). In years 2019 and 2020, germination rates were too poor, and consequently no planting occurred at the field sites. During this multi-year study, we observed that for all tested years, agretti’s seed viability was very poor beyond a storage period of three months. Hence, there was only one growing season per year because seed germination was too low.

For each planting, two soil samples were collected to a depth of 0-30 and 30-60 cm in each sub-plot (three one-meter sub-plots randomly located per bed) at preplant and at harvest for each respective planting and composited, respectively, for each bed. Table 1 shows soil chemical properties, including acid extractable Se concentrations for three different soil depths (0–30, 30–60, 60–90 cm) at pre-planting for each growing year. Soil samples were processed, as described later. Plants were field-grown under organic- like conditions without the use of synthetic chemicals, i.e., pesticides, herbicides, or fertilizers. Although the growing conditions were not certified as organic, because of the environmentally fragile conditions (water scarcity and saline soils), growers at Red Rock Ranch are required to follow California state guidelines similar to organic operation (CDFA, 2020).

**TABLE 1:** Soil chemical properties at field sites at pre-planting for growing seasons in 2016, 2017, 2018, and 2021, respectively. Values represent average (*n* = 3) ± SD.

				Water extractable								Acid Extractable^§^

Year	Depth	pH	EC	Cl	B	Ca	K	Mg	Na	S	Se	Se
	cm		mS/cm	—————————————-mg/L—————————————————————————————–								mg/kg DW
2016	0–30	7.99 ± 0.09	8.07 ± 1.59	510 ± 146	10.2 ± 2.2	488 ± 21	17.9 ± 2.4	60 ± 5	1614 ± 457	1390 ± 240	0.06 ± 0.03	1.6 ± 0.1
	30–60	8.14 ± 0.11	13.6 ± 0.95	937 ± 170	17.2 ± 1.4	466 ± 15	17.7 ± 1.4	73 ± 3	3372 ± 254	2365 ± 121	0.22 ± 0.07	1.5 ± 0.2
	60–90	8.15 ± 0.10	16.0 ± 1.25	1237 ± 187	18.7 ± 1.5	463 ± 14	19.6 ± 2.0	78 ± 8	4053 ± 346	2679 ± 188	0.29 ± 0.06	1.2 ± 0.1
2017	0–30	7.99 ± 0.21	6.99 ± 1.61	277 ± 145	9.2 ± 3.4	485 ± 15	13.0 ± 4.7	54 ± 8	1282 ± 410	1246 ± 271	0.12 ± 0.06	1.9 ± 0.2
	30–60	8.27 ± 0.11	13.0 ± 1.39	827 ± 246	18.6 ± 2.5	488 ± 29	11.6 ± 2.8	72 ± 5	2894 ± 336	2231 ± 196	0.34 ± 0.09	1.7 ± 0.4
	60–90	8.24 ± 0.17	16.5 ± 1.75	1360 ± 415	21.4 ± 3.6	504 ± 37	12.1 ± 4.3	85 ± 12	3766 ± 442	2731 ± 246	0.43 ± 0.16	1.5 ± 0.2
2018	0–30	8.12 ± 0.04	8.47 ± 1.32	529 ± 267	12.0 ± 2.1	316 ± 72	19.1 ± 4.1	102 ± 18	1743 ± 397	1212 ± 160	0.52 ± 0.13	3.4 ± 0.4
	30–60	8.29 ± 0.06	12.5 ± 0.96	951 ± 145	13.9 ± 1.1	237 ± 73	14.1 ± 6.0	128 ± 11	3045 ± 317	1871 ± 203	1.14 ± 0.25	2.2 ± 0.4
	60–90	8.30 ± 0.07	14.0 ± 0.98	1261 ± 283	12.4 ± 1.1	180 ± 64	14.8 ± 5.1	137 ± 13	3558 ± 333	2048 ± 222	0.85 ± 0.24	1.5 ± 0.2
2021	0–30	7.88 ± 0.06	10.33 ± 2.71	1582 ± 574	12.2 ± 4.0	563 ± 52	22.4 ± 7.9	110 ± 26	1825 ± 655	1122 ± 237	0.44 ± 0.12	1.1 ± 0.2
	30–60	8.26 ± 0.11	13.0 ± 0.87	1008 ± 152	16.0 ± 1.2	397 ± 42	11.3 ± 2.8	91 ± 9	3274 ± 281	2156 ± 192	0.56 ± 0.14	1.8 ± 0.2
	60–90	8.23 ± 0.91	15.5 ± 1.09	1286 ± 236	17.4 ± 1.3	449 ± 38	15.5 ± 3.2	100 ± 16	3792 ± 401	2486 ± 248	0.52 ± 0.09	1.4 ± 0.1

^§^Acid extractable Se in 2021 were estimated based upon pervious planting years.

Plants were grown during four different growing seasons each year due to the variation in seed viability and germination rates amongst the four years. The first planting occurred from 11 July to 21 August 2016; the second planting occurred from 23 May to 24 July 2017; the third planting occurred from 16 April to 25 June 2018; and the fourth planting occurred from 24 February to 26 April 2021. Plantings in 2016 and 2017 took place on the same field plot, while planting in 2018 and 2021 took place on a field site adjacent to field site used in the earlier plantings.

### Irrigation treatments

A surface-drip irrigation system was installed consisting of one in-line turbulent flow emitter per bed with an emitter spacing of 0.45 m and a flow rate of 4 L/h on the field site. Low-saline water (EC < 1.0 dS m^–1^) was sprinkled irrigated and applied at time of transplanting to promote initial establishment of plants. The amount of total irrigation water applied to microplots was based on rates of 100% evapotranspiration (Et_*o*_) (treatment “High”), 75% Et_*o*_ (treatment “Medium”), and 50% of Et_*o*_ (treatment “Low”), respectively. Irrigation amounts were determined by multiplying the average potential evapotranspiration (ET_*o*_) data recorded by CIMIS station #2 (California Irrigation Management Information System at Five Points/UC Westside Field Station) by forage crop coefficient (K_*c*_), averaging 0.35 (early season) to 1.15 (mid-season) to 0.75 (end of season). Crop coefficients were adjusted according to their respective growing season for each respective planting. Information on the total amount of water applied per irrigation water treatment (high, medium and low was based on ET_*o*_) is shown in Table 2. The experimental site was a completely randomized block design. Each irrigation treatment was replicated six times, with a replicate consisting of one bed (already described) (Supplementary Figure 1). Two types of water quality were used for irrigation for the multi-year study: low-saline water and saline water. Low-saline water solely was used to irrigate agretti in 2016 and 2017, and saline water only was used to irrigate agretti in 2018 and 2021. The saline water was collected from furrow irrigated field sites adjacent to the microplots. This source of water was collected and stored in a drainage pond reservoir adjacent to test field site. The saline water was then pumped, filtered, and utilized in the agretti field plot with the surface drip irrigation system. Saline water composition used for irrigation on the microplots generally had salinity levels ranging from 3 to 8 dS m^–1^, 4–8 mg B L^–1^ and 0.12–0.25 mg Se L^–1^ while low-saline water had salinity levels ranging from 0.2 to 0.7 dS m ^–1^, 1 mg B L^–1^ and 0.02 mg Se^–1^ (water quality characteristics are shown in Table 3).

**TABLE 2:** Total amounts of either low-saline or saline water applied at different rates (Et_o_ %) to microplots for all four years for planting seasons in 2016, 2017, 2018, and 2021, respectively.

		Treatment (Et_o_%)			
		
Year of planting	Days of irrigation	100%	75%	50%	Precipitation
		—————mm——————————			
2016	40	263	203	144	0
2017	62	485	363	249	1
2018	70	430	308	217	7
2021^†^	56	201	149	99	29

^†^Direct-seed planting took place in February of 2021 (see section materials and methods).

**TABLE 3:** Chemical characteristics of low-saline water and saline water applied to microplots for growing seasons in 2016, 2017, 2018, and 2021, respectively. Values represent average (*n* = 3) ± SD of water samples collected through respective growing season.

Water quality	Year	pH	EC	Cl	B	Ca	K	Mg	Na	S	Se
			mS/cm	——————————————-mg/L—————————————————————–							
Low-saline^‡^	2016	7.63 ± 0.12	2.95 ± 0.6	554 ± 66	3.5 ± 0.7	85 ± 31	2.1 ± 1.0	4.7 ± 2.2	624 ± 163	249 ± 57	0.06 ± 0.02
	2017	7.79 ± 0.5	3.43 ± 1.3	517 ± 84	4.4 ± 1.7	71 ± 24	2.4 ± 0.8	3.1 ± 1.4	761 ± 161	377 ± 266	0.08 ± 0.03
Saline	2018	8.37 ± 0.5	9.25 ± 2.4	1257 ± 351	16.3 ± 5.8	427 ± 137	5.8 ± 2.1	108 ± 39	1606 ± 505	903 ± 305	0.35 ± 0.14
	2021	8.01 ± 0.2	5.19 ± 0.3	1244 ± 240	5.1 ± 0.2	134 ± 42	5.6 ± 1.0	48 ± 6	770 ± 75	199 ± 11 5	0.01 ± 0.01

^‡^Waters were also tested for biological and pathogenic activities.

### Harvest

Plants were grown for 40 d, 61 d, 69 d, and 50 d^1^ for plantings in 2016, 2017, 2018, and 2021, respectively. Harvest for each year generally occurred after we visually determined that “agretti” shoots were still young and tender (preferred for human consumption), irrespective of water treatment. Above-ground young vegetative growth was cut 1 cm above the soil at soil sampling sites located in sub-plots within each bed. For analyzing data, plant biomass and yield of the single cuttings were measured on the three one-meter sub-plots (Supplementary Figure 1) randomly selected for each respective water treatment on each bed. Shoots samples were washed in deionized water, weighed, dried at 55–65*^o^*C for three days, weighed again, and ground with the Udy Cyclone Mill with a 1.0 mm screen. Twenty grams composite sub-samples of freshly harvested agretti from each respective planting were placed in chests filled with ice, transported to laboratory, and stored at −80°C for future analyses on Se speciation and total phenolics (described later). The other remaining plants on field site were harvested and donated to restaurants and farmers’ markets, which showed a strong interest in this new Se enriched halophytic vegetable (its potential marketability is discussed later). After harvest, soil samples were again collected at the same sub-plot locations sampled prior to transplanting, as already described. They were dried and processed, as already described for shoot material.

### Selenium extraction: Soluble and protease

To determine Se speciation in agretti shoot samples, sub-samples store at - 80°C were retrieved. Due to unexpected power outages and loss of electricity, all sample stored at -80°C were lost, except samples collected from planting (transplanted and direct-seeded) in 2021. For Se speciation the samples were processed as follows: methanol chloroform water (MCW) solvent extraction (described as “soluble” throughout text) and MCW enzymatic digest (with protease) were used to separate the soluble Se compounds (non-protein bound) and insoluble compounds (protein bound) for identification and quantification. The MCW extraction used 1 g of freeze-dried ground and sieved tissue sample added to 40 mL glass vials with a Teflon cap and separated in two sets of replicates (soluble and protease). Fifty mg of protease from Streptomyces griseus Type XIV (Sigma-Aldrich) was added to the protease replicates (Montes-Bayón et al., 2002), which hydrolizes peptide bonds, releasing Se amino acids into solution. Next, 10 mL of ultrapure water at room temperature were added to these vials containing protease. The other set of samples (soluble) received 17 mL of methanol (Optima grade) and contained no protease. The samples were vortexed, and the protease sample set was incubated in a shaker for 20 h at 37°C, while the methanol only sample set was placed overnight at 4°C. After digestion, 17 mL of methanol were added to the protease samples (to denature the protease enzyme and stop enzymatic activity), and 10 mL of ultrapure water were added to the soluble digested methanol extractions. Each tube was vortexed multiple times and refrigerated overnight at 4°C. Following this, 8.5 mL of chloroform (Optimal grade) were added to all vials and capped, shaken vigorously, and refrigerated at 4°C overnight until the tissue was fully extracted, and the upper aqueous (methanol-water) phase had fully partitioned from the chloroform phase. The upper aqueous (methanol-water) containing the extracted Se compounds was removed and transferred to a centrifuge tube. One quarter of the aqueous (methanol-water) phase was then pipetted into 50 mL ICP digestion tubes for drying, acid digestion, and analysis of total aqueous Se by ICP-MS (described later). The fully extracted tissue remaining in the chloroform phase was then dried, acid digested, and analyzed for total Se by ICP-MS. The Se extraction efficiency (80%) in the aqueous phase (soluble and protease extracts) was calculated from these ICP-MS results as: (total Se in methanol-water phase) / [(total Se in methanol-water phase) + (total Se in chloroform phase)] × 100. The remaining aqueous (methanol-water) phase was dried in vacuum at -140°C by refrigerated centrifugal speed vacuum (Labconco CentriVap Concentrator), re-suspended to 2.5 mL with ultrapure water, and stored in a −80°C freezer. Final clean-up of the concentrate used Waters Sep-Pak Classic C18 cartridge (360 mg 55–105 μm). Each cartridge was cleaned by flushing 10 mL of methanol and 5 mL ultrapure water in succession. The 2.5 mL concentrates were thawed, vortexed, and 11 μL of 88 % formic acid (ACS grade, Fisher Chemical) were added prior to being transferred by disposable Pasture pipette to the Sep-Pak. The column was loaded with the sample, which was pushed through, and soluble residual was eluted with 3 mL methanol. The total eluent (methanol-water) was collected into a 50 mL conical tube and then dried completely using refrigerated centrifugal speed vacuum. This dry extract pellet was then re-suspended in 1.5 mL ultrapure water and centrifuged in Corning Costar Spin-X centrifuge tube filters (0.22 μm at 10,000 rpm). The filtered samples were then transferred into Agilent 2 mL screw top glass vials with septa and frozen until SAX-HPLC-ICP-MS analysis.

### Selenium speciation and total Se analyses

The Se speciation analysis (organic and inorganic Se) of the soluble and protease extracts from agretti is described in detail by Bañuelos et al. (2012). Selenium speciation analyses used an Agilent 1200 HPLC equipped with a Hamilton PRP-X100 strong anion exchange column (10 μm particle size 250 mm length and 4.1 mm internal diameter) coupled to the Agilent 7500 CX ICP-MS (SAX-HPLC-ICP-MS). The ICP-MS was equipped with a quadrupole detector and an Octopole Reaction System (ORS) utilizing hydrogen as a cell gas (5.5 mL/min) to minimize Se polyatomic interferences. Dried ground agretti was analyzed for total Se concentrations by Agilent 7500 CX ICP-MS (Agilent Technologies Santa Clara, USA) and other elements with the inductively coupled plasma optical emission spectrometry (ICP-OES) (Varian Vista-Pro Santa Clara, CA, USA) after wet-acid digestion with HNO_3_, and H_2_O_2_, and HCl. Generally, for Se speciation a single analysis (30 μL injection) was conducted for each of the broths, soluble, and protease extract replicates (*n* = 3). Chromatographic separation of Se was achieved with an isocratic mobile phase of 5 mM ammonium citrate buffer (pH 5.2) with 2% methanol at flow rate of 1 mL/min. The two instruments (Agilent 1200 HPLC and Agilent 7500 CX ICP-MS) were integrated through Agilent Chemstation software with chromatographic data analysis. The retention times of Se-78 containing peaks were monitored using the ICP-MS and directly compared to the authentic standard (listed below), retention times, and secondary confirmation by spiking samples with standards to account for any matrix induced changes to the chromatographic analysis, as described by Bañuelos et al. (2012). The SAX-HPLC-ICP-MS standards utilized included sodium selenate (Na_2_SeO_4_), sodium selenite (Na_2_SeO_3_), SeMet, and SeCys_2_ (all purchased from Sigma-Aldrich, St. Louis, MO). Additionally, methyl-selenocysteine (MeSeCys), selenocystathionine (SeCyst), and γ-glutamyl-methyl-selenocysteine (γ-gluMeSeCys) were all purchased from Pharma Se.

### Quality control for Se and Se speciation

The National Institute of Standards and Technology (NIST) wheat flour (SRM 1567a) was used as the standardized quality control for wet-acid digestion (total Se concentration) and Se speciation extraction (SeMet, SeCys_2_) content in plant material. The SRM 1567a was utilized as an internal control in the MCW extraction to account for any changes in the protease XIV efficacy and other factors during extraction process. The total Se recovery rates were over 94 % for the wheat flour standard, which has a Se concentration of 1.1 ± 0.2 μg Se/g DW, with a method detection limit of 50 μg Se/g DW. The selenoamino acid content in SRM 1567a consisted of 92% SeMet and 6% SeCys_2_. The NIST wheat flour standard was always included in triplicate with each plant powder and respective agretti sample. Overall, Se speciation extraction efficacy, including MCW (soluble; free and unbound Se) and protease extractable Se (protein bound Se) was at least 80% for agretti, and wheat standard matrixes. The extraction and quality control measures are documented in detail (Bañuelos et al., 2012).

### Total phenolics

Total phenolic concentrations were measured in stored agretti samples from planting 2021 (described earlier) according to (Singleton et al., 1998) using the Folin-Ciocalteu reagent assay. Absorbance was measured at 756 nm using a Spectra Max plus 384 spectrophotometer (Molecular Devices, Sunnydale, CA). Total phenols concentration was standardized against gallic acid (GA) and expressed as milligram of gallic acid equivalents (GAE) per L of fruit juice. The linearity range for this assay was determined as 50-250 mg/L GA, giving absorbance range of 0.5–2.55 AU. The total phenolic analyses is used as an indicator of plant stress.

### Statistical analysis

All data were analyzed using Sigmaplot version 14.5. We tested significance and pairwise comparison amongst the irrigation treatment (levels of Et_*o*_) in each planting year because plants were grown during four different growing seasons. Significance was set at the 5% level. Data have been log transformed when they were not normally distributed. There were about 5–8% outliers that have been averaged out with the non-outliers within the same replication group. Statistical data analysis was performed with Gretl [Gnu Regression, Econometrics and Time-series Library, (Baiocchi and Distaso, 2003)].

## Results

### Plant growth

**FIGURE 1:**
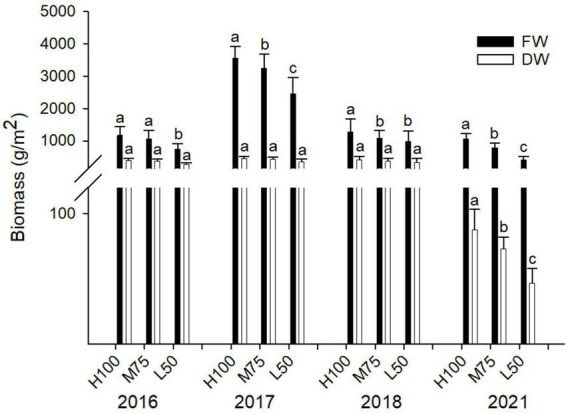
Fresh and dry weight of plant biomass in agretti grown for 4 years on saline, B- and Se-laden soil and irrigated with either low-saline water (2016 and 2017) or saline water (2018 and 2021). Statistical difference was performed to analyze the effect of irrigation levels within each planting year (H100 = high 100% Et_*o*_, M75 = medium 75% Et_*o*_, and L50 = low 50% Et_*o*_) as described in Table 3. Values represent averages (*n* = 12 for 100% Et_*o*_, *n* = 9 for 75% and 50% Et_*o*_ for planting in 2016; *n* = 19 for 100% Et_*o*_, *n* = 17 for 75% for planting in 2017; *n* = 18 for planting in 2018, *n* = 4 for planting in 2021). Values of *n* varies amongst year because of the variability in seed germination and plantlets survival in the field. and error bars represent standard deviation. Different letters indicate that values differed significantly (*P* ≤ 0.05), ns = not significant.

In this study, no plant toxicity symptoms were observed for any planting; irrespective of water quality applied or year of planting. Results of fresh and dry weight of agretti are shown in Figure 1 for all four years. Fresh weight yields were highest in 2017 (plants were irrigated with low-saline water and growing season was May thru July) compared to the different seasons in other planting years. Overall, fresh biomass was significantly higher when the plants received water of either quality at 100% Et_*o*_ compared to 75 and 50% Et_*o*_ for all plantings, except for 2016, when yield at 100 and 75% Et_*o*_ were similar. In 2016, 2017, and 2018, dry weight was not affected by the rate of irrigation water applied, and the values of dry weight were comparable across treatments (Figure 1). In 2021, the dry weight was significantly lower than in previous years (Note: 2021, yields are presented from plants that were direct seeded). The amount of irrigation water applied affected dry weight, causing a significantly lower dry weight at 50% Et_*o*_ compared to 100 and 75% Et_*o*_ treatments.

### Plant nutrients

Among the plant macronutrients some effects from irrigation rates and quality were observed. Magnesium was significantly higher when plants received 75 and 50 % Et_*o*_ in 2021 and in 2018, when plants received 50% Et_*o*_ (Supplementary Table 1) with saline water. P was significantly higher in 2016 and 2017, when the plants were irrigated with low-saline water and S was significantly higher in 2017 (when irrigated with low-saline water), and in 2018 when plants were irrigated with saline water (Supplementary Table 1). The micronutrients, Fe, Mn, and Zn were significantly higher in 2021 with irrigation with saline water but Cu was significantly lower (Supplementary Table 2). Fe was higher in 2021 when the plants were irrigated with 75% of Et_*o*_ and generally, Cu, Mn, and Zn were only affected by the planting year and not the water treatment (Supplementary Table 2).

### Selenium

**FIGURE 2:**
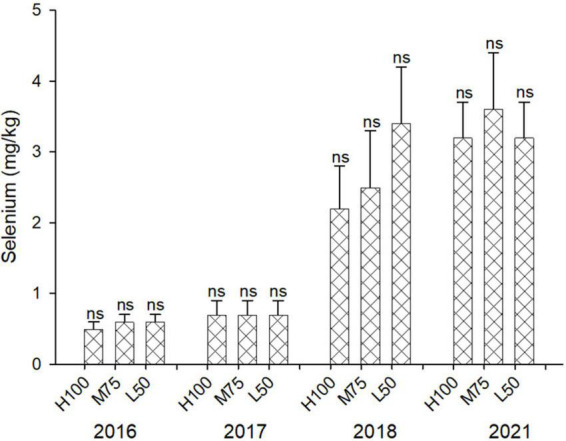
Concentration (mg/kg DW) of Se in plants of agretti grown for 4 years on saline, B- and Se-laden soil and irrigated at different rates (Et_*o*_ %) with either low-saline water (2016 and 2017) or saline water (2018 and 2021). Statistical difference was performed to analyze the effect of irrigation levels within each planting year (H100 = high 100% Et_*o*_, M75 = medium 75% Et_*o*_, and L50 = low 50%Et_*o*_), as described in Table 3. Values represent average (*n* = 12 for 100% Et_*o*_, *n* = 9 for 75% and 50% Et_*o*_ for planting in 2016; *n* = 19 for 100% Et_*o*_, *n* = 17 for 75% for planting in 2017; *n* = 18 for planting in 2018, *n* = 4 for planting in 2021, direct seeding) and error bars represent standard deviation. There was no significance amongst any treatments.

The concentration of Se in agretti ranged from 0.2 to 0.7 mg/kg DW in 2016 and 2017 with low-saline irrigation water, and 2.1 to 3.6 mg/kg DW in 2018 and 2021, respectively, with saline irrigation water (Figure 2). There was no significant effect of irrigation Et_*o*_% treatment on Se accumulation in shoots for any year, irrespective of water quality. Our results indicate that agretti is able to accumulate high levels of Se in its edible biomass when grown in Se-rich soil and irrigated with either saline or low-saline water. We observed, however, higher concentrations of tissue Se when irrigating with Se-containing saline water.

**FIGURE 3:**
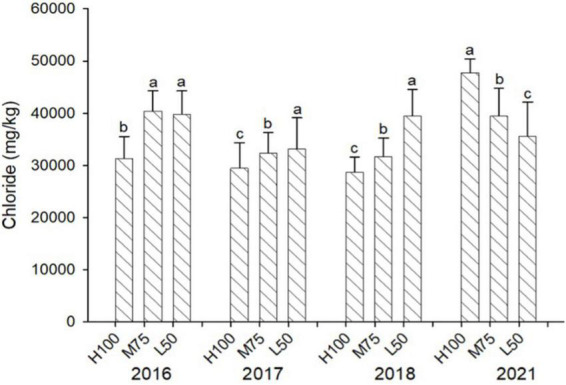
Concentration (mg/kg DW) of Cl in plants of agretti grown for 4 years on saline, B- and Se-laden soil and irrigated at different rates (Et_*o*_%) with either low-saline water (2016 and 2017) or saline water (2018 and 2021). Statistical difference was performed to analyze the effect of irrigation levels within each planting year (H100 = high 100% Et_*o*_, M75 = medium 75% Et_*o*_, and L50 = low 50% Et_*o*_) as described in Table 3. Values represent average (*n* = 12 for 100% Et_*o*_, *n* = 9 for 75% and 50% Et_*o*_ for planting in 2016; *n* = 19 for 100% Et_*o*_, *n* = 17 for 75% for planting in 2017; *n* = 18 for planting in 2018, *n* = 4 for planting in 2021, direct seeding) and error bars represent standard deviation. Different letters indicate that values differed significantly (*P* ≤ 0.05), ns = not significant.

**FIGURE 4:**
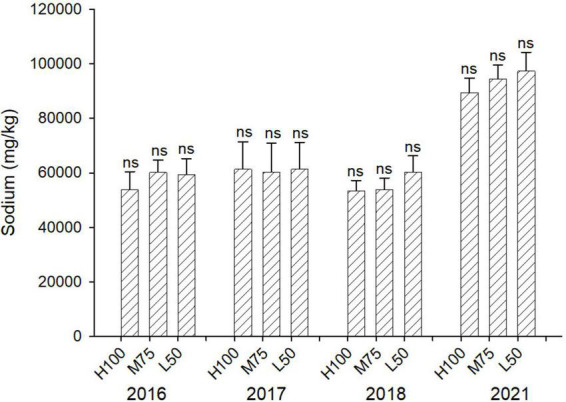
Concentration (mg/kg DW) of Na in plants of agretti grown for 4 years on saline, B- and Se-laden soil and irrigated at different rates (Et*_*o*_*%) with either low-saline water (2016 and 2017) or saline water (2018 and 2021). Statistical difference was performed to analyze the effect of irrigation levels within each planting year (H100 = high 100% Et_*o*_, M75 = medium 75% Et_*o*_, and L50 = low 50% Et_*o*_) as described in Table 3. Values represent average (*n* = 12 for 100% Et_*o*_, *n* = 9 for 75% and 50% Et_*o*_ for planting in 2016; *n* = 19 for 100% Et_*o*_, *n* = 17 for 75% for planting in 2017; *n* = 18 for planting in 2018, *n* = 4 for planting in 2021, direct seeding) and error bars represent standard deviation. Different letters indicate that values differed significantly (*P* ≤ 0.05), ns = not significant.

Figures 3, 4 show concentrations (mg/kg DW) of Cl and Na in harvested dried plant material. Na and Cl concentrations in shoots were >2% for Cl and >5% for Na, indicating that agretti, being a halophyte, accumulates high levels of these salt ions in its shoots, irrespective of saline or low-saline irrigation. The concentration of Cl was significantly higher in the 75 and 50% Et_*o*_ treatments compared to 100% Et_*o*_ in 2016, 2017, and 2018. In 2021, the concentration of Cl was, however, significantly higher at 100% Et_*o*_ compared to 75 and 50% Et_*o*_ (Figure 3). There were no significant differences in Na concentrations in shoots across treatments and years, and the concentration of Na was higher in 2021 (8–10% DW) compared to previous years (3–5 % DW) (Figure 4); In 2016 and 2017, the plants were irrigated with low-saline water compared to irrigation with saline water in 2018 and 2021. The different irrigation water quality did not strongly affect Na and Cl accumulation in shoot (Figures 3, 4). Concentrations of both ions were similar in 2016, 2017, and 2018, since the plants were grown in saline soil for all treatments every year, irrespective of water quality applied via irrigation.

Concentrations of B are similar across all water treatments (ET_*o*_) (Figuref 5), and the addition at B applied with saline water in 2018 (16 mg B/L) did not result in significantly different plant concentrations of B compared to 2016 and 2017. However, the concentration of B in agretti in 2021 was lower than in the previous years (2016–2018). This indicates that direct-seed planting from seeds may increase the ability of agretti to tolerate higher B concentrations, if they accumulated lower concentrations of B.

**FIGURE 5:**
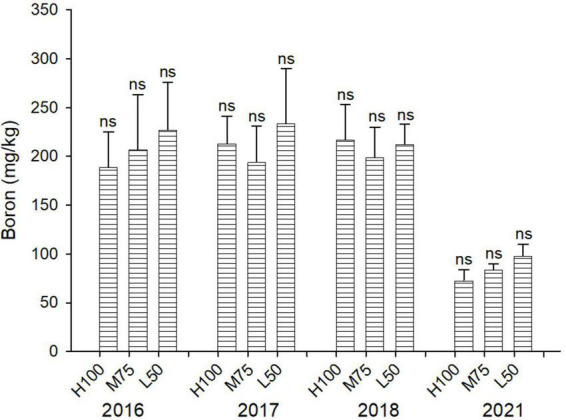
Concentration (mg/kg DW) of B in plants of agretti grown for 4 years on saline, B- and Se-laden soil and irrigated at different rates (Et_*o*_%) with either low-saline water (2016 and 2017) or saline water (2018 and 2021). Statistical difference was performed to analyze the effect of irrigation levels within each planting year (H100 = high 100% Et_*o*_, M75 = medium 75% Et_*o*_, and L50 = low 50% Et_*o*_) as described in Table 3. Values represent average (*n* = 12 for 100% Et_o_, *n* = 9 for 75% and 50% Et_o_ for planting in 2016; *n* = 19 for 100% Et_o_, *n* = 17 for 75% for planting in 2017; *n* = 18 for planting in 2018, *n* = 4 for planting in 2021, direct seeding) and error bars represent standard deviation. Different letters indicate that values differed significantly (*P* ≤ 0.05), ns = not significant.

### Selenium speciation and total phenolics

**FIGURE 6:**
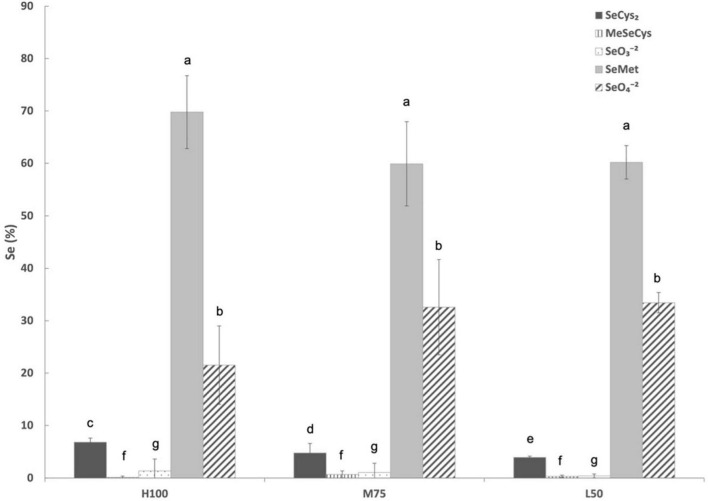
Selenium speciation in direct seeded agretti grown in 2021 and irrigated with saline waters at different rates. Statistical difference was performed to analyze the effect of irrigation levels within each planting year (H100 = high 100% Et_o_, M75 = medium 75% Et_o_, and L50 = low 50% Et_o_) as described in Table 3. Values represent the mean (*n* = 3) and error bars represent standard deviation. Different letters indicate that values differed significantly (*P* ≤ 0.05), ns = not significant. SeCys_2_: Selenocysteine; MeSeCys: Methylselenocysteine; SeO_3_^– 2^: Selenite; SeMet: Selenomethionine; SeO_4_^– 2^: Selenate.

Unfortunately, only tissues samples collected in 2021 were analyzed for Se speciation and total phenolics (as previously mentioned stored agretti samples at –80°C from 2017, 2018, and 2019 were lost) due to electrical power outage. Figure 6 shows Se speciation in agretti planted directly by seed in 2021. Irrespective of water treatment, SeMet was always the predominate Se species (between 60 and 70%), followed by SeO_4_^–2^ (20-32%) and then SeCys_2_ (2–5%). There were no significant effects of irrigation treatment (Et_*o*_%) for all Se species, except for SeCys_2,_ which showed a significantly higher concentration with high irrigation treatment (100% Et_*o*_ > 75% Et_*o*_ > 50% Et_*o*_).

Limited phenolic data (as described in the section materials and methods) showed values that did not exhibit any significant stress (indicated by total phenolic content) for agretti grown as direct seeded for any irrigation treatments. Total phenolics ranged from 180 GA mg/L (at highest treatment of 100% Et_*o*_) to a high of 257 GA mg/L (at lowest water treatment of 50% Et_*o*_).

### Soil analyses

Soluble soil chemical properties are shown at postharvest for all four years in Table 4. Averaged over four years, soil salinity ranged from a low of 3 to a high of 14 dS m^−1^ (Table 4), soluble B ranged from a low of 3 to a high of 19 mg/L, and soluble Se ranged from a low of 30 to a high of >1000 μg/L. These levels of salinity and B are considered toxic to most agronomic crops (Grieve et al., 2011). Soil salinity, soluble B and Se concentrations were significantly higher at the deeper depths (30–60 cm) after harvest. Generally. Soil EC was greater with 50 and 75% Et_*o*_ treatments. Irrigation at 100% Et_*o*_ likely induced some leaching of salinity compared to lower Et_*o*_ treatments.

**TABLE 4:** Soil concentration of Cl, Na, B, and Se, and levels of EC at field site irrigated with different rates (irrigation treatment Et_o_) in 4 years at post-harvest of agretti at two soil depths. Values represent average (*n* = 5) ± SD.

Year		Soil depth	EC	Cl	B	Na	Se	Se

Water extractable								Acid extractable^§^
	
	% Et_o_	cm	mS/cm	———————-mg/L—————–				mg/kg
2016^+^	100	0–30	6.3 ± 1.3	266 ± 82	6.7 ± 2.1	1101 ± 356	0.06 ± 0.01	1.9 ± 0.2
	100	30–60	13.6 ± 1.4	916 ± 230	18.0 ± 3.4	3117 ± 317	0.24 ± 0.07	1.9 ± 0.3
	75	0–30	7.6 ± 1.6	429 ± 152	10.5 ± 2.5	1453 ± 458	0.09 ± 0.05	1.9 ± 0.1
	75	30–60	14.0 ± 1.4	1049 ± 363	18.2 ± 2.4	3185 ± 380	0.29 ± 0.15	1.8 ± 0.2
	50	0–30	8.86 ± 2.5	521 ± 285	11.2 ± 3.1	1808 ± 705	0.11 ± 0.08	1.8 ± 0.2
	50	30–60	12.9 ± 3.5	903 ± 389	17.9 ± 2.9	2933 ± 964	0.22 ± 0.10	1.7 ± 0.2
2017^+^	100	0–30	4.6 ± 1.4	71 ± 44	5.3 ± 3.4	606 ± 340	0.03 ± 0.02	1.7 ± 0.1
	100	30–60	10.3 ± 1.7	312 ± 212	16.4 ± 3.1	2123 ± 461	0.12 ± 0.07	1.6 ± 0.1
	75	0–30	3.8 ± 0.5	54 ± 34	3.4 ± 1.2	430 ± 132	0.03 ± 0.01	1.7 ± 0.2
	75	30–60	10.5 ± 1.6	335 ± 167	15.6 ± 2.9	2253 ± 447	0.12 ± 0.07	1.6 ± 0.3
	50	0–30	4.74 ± 1.0	80 ± 48	5.0 ± 2.0	662 ± 224	0.03 ± 0.02	1.7 ± 0.2
	50	30–60	12.5 ± 2.2	602 ± 238	19.7 ± 4.0	2759 ± 605	0.2 ± 0.11	1.7 ± 0.2
2018^‡^	100	0–30	8.1 ± 1.9	441 ± 227	11.9 ± 3.9	1543 ± 588	0.27 ± 0.20	3.4 ± 0.3
	100	30–60	12.0 ± 1.5	779 ± 339	16.3 ± 1.7	2726 ± 511	0.89 ± 0.66	2.0 ± 0.4
	75	0–30	9.7 ± 3.1	665 ± 516	14.0 ± 3.9	1959 ± 940	0.34 ± 0.26	2.8 ± 0.6
	75	30–60	13.0 ± 3.6	979 ± 718	16.3 ± 3.3	2891 ± 918	0.8 ± 0.51	1.9 ± 0.1
	50	0–30	9.0 ± 1.0	537 ± 206	14.3 ± 2.2	1709 ± 291	0.32 ± 0.22	3.2 ± 0.3
	50	30–60	14.5 ± 1.6	1345 ± 303	18.0 ± 2.9	3272 ± 539	1.32 ± 0.21	2.5 ± 0.6
2021^‡^	100	0–30	8.97 ± 1.4	834 ± 187	11.2 ± 1.5	1552 ± 335	0.23 ± 0.04	2.3 ± 0.2
	100	30–60	14.43 ± 2.4	1343 ± 420	16.9 ± 2.0	2930 ± 655	0.65 ± 0.07	1.8 ± 0.1
	75	0–30	9.20 ± 0.86	858 ± 128	12.6 ± 2.7	18.52 ± 366	0.22 ± 0.08	2.1 ± 0.2
	75	30–60	12.52 ± 1.8	1122 ± 105	15.9 ± 2.0	2417 ± 457	0.53 ± 0.09	1.7 ± 0.1
	50	0–30	9.77 ± 1.0	959 ± 170	13.7 ± 1.1	1684 ± 280	0.28 ± 0.13	2.2 ± 0.2
	50	30–60	13.36 ± 0.78	1203 ± 106	15.7 ± 2.1	2630 ± 226	0.79 ± 0.16	2.0 ± 0.1

^+^Values represent irrigated with low-saline water.

^‡^Values represent irrigated with saline water.

^§^Acid extractable Se in 2021 were estimated based upon pervious planting years.

## Discussion

This study is a first field investigation on the feasibility of growing agretti under organic- like growing conditions as a Se-biofortified crop under saline and B-irrigated field conditions in the westside of Central California. Previous studies have identified agretti as a potential halophyte crop for growing in saline conditions (Colla et al., 2006; Calone et al., 2021), as well as in in saline/Se-rich soils and irrigating with Se-enriched water under greenhouse conditions (Centofanti and Bañuelos, 2015; Zhu et al., 2019). In this field study, agretti was grown on Se-rich soil and irrigated with either low -saline water or with saline water naturally enriched with Se and B at different irrigation rates (% of ET_*o*_). The natural occurrence of Se in the soil and the additional contribution of Se via saline water application, resulted in a high accumulation of Se, irrespective of water application rate. Growing agretti in a Se rich soil and irrigating with saline water appears to be a natural strategy for more effectively producing Se biofortified agretti under these arid growing conditions. The mean concentration of tissue Se in agretti with low-saline irrigation water was 640 μg Se kg^–1^ DW and 2940 μg Se kg^–1^ DW with saline irrigation. If a serving portion (100 g fresh agretti material, corresponding to about 30 g DW) was consumed, then agretti (with tissue Se concentrations presented in Figure 2) could provide 19.2 μg Se/serving when harvested from low-saline irrigation and 88.2 μg Se/serving when harvested from saline irrigation, on these saline/Se-laden soils, respectively. The average required level of Se in the human diet is 50–55 μg/day Se (Gupta, 2020). Although the window between toxicity and deficiency is narrow (∼ 400 μg Se/day vs ∼ 40 μg Se/day), Se deficiency is more widespread than Se toxicity (Coppinger and Diamond, 2001; Broadley et al., 2006; White and Broadley, 2009). Since plants are the main source of Se for most humans and livestock across the world, we have demonstrated that Se biofortification is possible with agretti when irrigated with low-saline or saline water in the westside of Central California.

Our results do not show a clear trend of increased Se accumulation in agretti under water deficit irrigation, i.e., 50% Et_*o*_. Thus, it appears that water application rate of either low-saline or saline water does not significantly affect Se accumulation in agretti grown in saline soils. Selenium accumulation in plants grown under saline growing conditions may be advantageous for the plant, because Se accumulation in plants may affect other physiological processes within plants related to increasing salt tolerance. In this regard, others have shown that accumulated Se in plants participates in antioxidant defense systems and it may enhance tolerance to abiotic stresses (Andrade et al., 2018), such as water deficit or excessive salinity. Djanaguiraman et al. (2005) reported that Se may play an important role in the adjustment of plant water status under drought stress and improve plant–water relations by lowering the osmotic potential of seedlings growing under water stress (Hartikainen, 2005; Nawaz et al., 2012). In addition to the role of Se potentially enhancing stress tolerance, halophytes, including Salsoda species, are already adaptive to tolerating high salinity (Shuyskaya et al., 2017; Hasanuzzaman et al., 2019; Mukhtar et al., 2019). Hence, the accumulation of Se may provide agretti with additional tolerance to high salinity.

The speciation of Se in agretti shoots in 2021 showed that for all irrigation treatments, SeMet was the predominant selenoamino acid, irrespective of planting method or any water treatment (i.e., application rate). Moreover, water application (ET_*o*_%) had no effect on Se speciation, except for SeCys_2_. Inexplicable effects from irrigation treatment were observed in SeCys_2_. We are currently investigating this irrigation effect on Se accumulation in other crops, e.g., tomatoes, to determine if irrigation with saline water influences Se speciation, a component important to understand in any biofortification strategy. Like most Se speciation identified in non-Se accumulator plant species, SeMet Is the predominant selenoamino acid in agretti biofortified with Se under natural growing conditions. Thus, consumption of Se-enriched agretti should increase Se intake by consumers.

In addition to being a potential Se-biofortification crop, agretti confirmed its salt tolerance (Calone et al., 2021) as well as B tolerance. Fresh and dry weight were not strongly affected by the presence of B and salts in the soil and saline irrigation water. The reasons for the higher fresh weight yields in 2017 may be related to combination of factors such as the planting date (May 23 to July 24) and the consequent different air temperatures (Supplementary Figure 2), the amount of water applied in each year, and the effect of irrigation water quality (low-saline versus saline water). Plant stress, also indicated by changes in total phenolic content, did not significantly differ among water treatments (Et_*o*_ %) in 2021. In this study, yields difference among the plants grown are not an accurate indication of salt stress, since harvest time for each respective growing season was virtually determined by the apparent tenderness of the edible shoot. This parameter of harvesting only tender shoots is important because consumers prefer eating young shoots and not shoots from older plants that are slightly more woody (Lone, unpublished). Consequently, yields are controlled strongly by the growers’ self-determination of harvest date. We also observed in 2021 that planting directly by seed resulted in a more tender shoot (less stem-like material) a physical characteristic that is more desirable for human consumption, especially for consumers in the Mediterranean region (Renna and Gonnella, 2020; Lombardi et al., 2022). Importantly there was no wood-like stem, which is common physical trait when harvesting transplanted agretti. Consequently, there were more pronounced differences fresh and dry weight biomass in 2021. This result is likely because plants grown by direct seeding in 2021 contained a higher water content in shoots- there was less stem and more agretti-like leaves.

Levels of accumulated B in shoot of agretti were comparable to those reported by Zhu et al. (2019), who grew agretti in hydroponic system under controlled conditions with solution B concentrations similar to those applied in this study with saline water. In our field study, B concentration in shoots was lower in 2021 compared to previous years. This effect is likely due to high salinity inhibition on inhibiting B uptake (shown in Figure 4 in 2021). Others have observed this effect of salinity on B uptake in other crops (Yermiyahu et al., 2008; Zhu and Bañuelos, 2016). However, direct-seeded agretti versus transplanted agretti may have inexplicitly also played a role in restricting B accumulation in the agretti shoots; hence enhancing agretti’s B tolerance. Thus, our results indicate that direct-seeded agretti may protect itself from excessive B in the soil and irrigation water by limiting its B uptake. Reducing B accumulation can be a plant defense-like response to excessive B in the root zone is imperative for any crop considered for growing in soil or with irrigation water containing B. Boron in irrigation water is toxic to typical agronomic crops, as described in Reid et al. (2004), Reid and Fitzpatrick (2009), at concentrations greater than 4 mg B/L.

Developing a Se-biofortification strategy with a saline water reuse system in the westside of SJV in Central California, importantly requires the identification of a cropping system with high salt and B tolerance and selecting a crop that has economic value for the growers. In this study, we have shown that producing Se-enriched agretti under organic- like growing conditions successfully produces a viable crop.

It is important to note the presence of high levels of Na in the shoots (5 to 9.8% DW) when growing Se-biofortified agretti under these tested saline growing conditions. Consequently, consumption of agretti produced under saline growing conditions should be monitored for people requiring a low Na diet. Further studies may explore the double potential of dried agretti biomass as Se-enriched food and as organic salt replacement, thus increasing the economic value of the plant.

Currently, studies on consumers’ acceptance and market viability of Se-enriched agretti are being carried out in Central California (Lone, unpublished). Preliminary results indicate that consumers will need more education about Se-enriched halophyte plants and the safety on using saline irrigation on food crops. Others have reported on the importance of having consumer acceptance of Se-enriched food products (Cox and Bastiaans, 2007; Wortmann et al., 2018). This acceptance is applicable for Se-biofortified agretti produced from saline growing conditions since a typical consumer is not aware of saline irrigation practices.

Most of the risks associated with the reuse of Se-enriched saline-sodic waters are related to degrading soil quality and to paying close attention to Se content in edible plant tissue (Imoff, 1991; Oster and Grattan, 2002; Grattan et al., 2014). Moreover, levels of other trace elements naturally present in westside soils of the SJV should also be closely monitored (Grattan and Oster, 2003; Suyama et al., 2007). To reduce the impact on the environment and human and livestock health, the sustained use of saline water for biofortifying crops with Se requires the implementation of special management practices, such as the biological management of salts, i.e., Na, by selecting salt tolerant agretti as a companion crop for Na removal (Colla et al., 2006) and the adoption of irrigation management practices when using saline water.

## Conclusion

This study identified a Se-biofortification strategy with the production of agretti using saline, B- and Se-laden soil and irrigating with saline and low-saline water, respectively. This is one of the first investigations on growing agretti as a Se-biofortified crop under organic-like field agriculture practices and irrigating with different amounts of low-saline and saline water in the west side of the SJV in California. To our knowledge, there is no information available on both producing Se-biofortified agretti or on production with irrigation of low-saline or saline water under high saline and B growing conditions.

There is a potential for the producing of Se-enriched agretti at in the saline soils of the SJV and similar arid areas with similar geological sources of Se. Because the sustainability of producing typical agronomic crops in California is decreasing due to a lack of good quality water, alternative salt and B tolerant crops need to be identified to accumulate Se, despite both excessive salts in soil and irrigation water. It is important that selected Se-biofortified crops like agretti have economic value and have farmers who will accept growing a new crop. In this regard, previous studies have shown a positive response to the marketability and consumption of agretti by the retail industry, gastronomy, and consumers (Lone, unpublished). The feasibility of large-scale production of Se-enriched, agretti depends on improving agronomic practices such as improved seed viability, germination potential, and optimal growth conditions. Importantly, consumer acceptance for consuming new Se-enriched crops produced from saline waters must also be take into consideration. For example, surveys conducted by Lone et al. (unpublished) indicate approximately three quarters of respondents have ‘no knowledge’ of halophyte plants such as agretti and 77.4% are not aware halophytes are food. When shown photos of agretti, only a small proportion of respondents know about the crop, but 92.7% are willing to try it, and 76.7% want it offered where they purchase food. When queried about irrigation water, 55.6% had ‘some knowledge’ about drainage and poor quality water irrigation, but only 13.7% were aware that saline drainage water can be used for irrigation of food crops. This general lack of knowledge about growing conditions may stem from consumers not fully understanding terms such as ‘saline,’ ‘non-saline,’ and ‘drainage water’ that were defined and used in the survey. Thus, marketers should be cognizant that additional consumer education and use of ‘consumer friendly’ terminology may be necessary when introducing new Se-enriched food products in the marketplace.

## Data availability statement

The raw data supporting the conclusions of this article will be made available by the authors, without undue reservation.

## Author contributions

GB: conceptualization, methodology, reviewing, and editing. TC: writing – original draft preparation, methodology, and data analysis. MZ: assisting with data curation. KV and TL: reviewing and editing. All authors contributed to the article and approved the submitted version.

## Footnotes

1 Fifty days was selected for harvest in 2021 because the plants looked more consumers’ friendly (e.g., young tender and soft tissues). These characteristics were more noticeable in direct-seeded plants.
