# Supplementary material for: Salsola soda as selenium biofortification crop under high saline and boron growing conditions
Source: Front Plant Sci. 2022 Sep 26;13:996502. doi: 10.3389/fpls.2022.996502 (PMC9549694; doi:10.3389/fpls.2022.996502)
Supplement: Supplementary file 1 [file Data_Sheet_1.docx]

**Potential of alternative crops for selenium biofortification under high saline and boron growing conditions: the case of *Salsola soda*.**

Gary S. Bañuelos^a*^, Tiziana Centofanti^b^, Clemencia Zambrano^b^, Todd Lone^c^, Kaomine Vang^b^

^a^ USDA, Agricultural Research Service, San Joaquin Valley Agricultural Science Center, 9611 South Riverbend Avenue, Parlier, CA 93648-9757 USA)

^b^ Center for Irrigation Technology, California State University Fresno, Fresno, CA 93740 USA

^c^ Department of Agricultural Business, Jordan College of Agricultural Sciences and Technology, California State University Fresno, Fresno, CA 93740 USA

*Corresponding author: Gary S. Bañuelos^a^

Phone: +1-559-596-2880

Email: gary.banuelos@usda.gov

**Supplementary material**

**Fig. 1S.** Micro-plots layout with location of the planted (vegetated) areas and irrigation system at Red Rock Ranch, Five Points, CA**.**

**Fig. 2S.** Climatic data for all four years if plant growth in the field.

Table 1S. Concentration of selected macronutrients in shoots of agretti grown for 4 years on saline, B- and Se-laden soil and irrigated with either non-saline water (2016 and 2017) or saline drainage water (2018 and 2021). Statistical difference was performed to analyze the effect of irrigation levels as described in Table 3. ns, *, **, and *** indicate non-significance or significance at 5%, 1%, and 0.1% probability levels, respectively. Y=year, and T= irrigation treatment (%Et_c_).

| Year | Irrigation treatment | Ca | | K | Mg | P | S |
| --- | --- | --- | --- | --- | --- | --- | --- |
|  | % Et_c_ |  | ----------------------g/kg----------------- | | | | |
| 2016 | 100^a^ | 6.1 ± 1.3 | | 11.0 ± 1.7 | 1.1 ± 0.1 | 1.9 ± 0.2 | 2.2 ± 0.3 |
|  | 75^b^ | 5.4 ± 0.7 | | 11.8 ± 2.6 | 1.1 ± 0.1 | 1.9 ± 0.3 | 2.5 ± 0.2 |
|  | 50^b^ | 6.4 ± 1.2 | | 11.8 ± 1.8 | 1.2 ± 0.1 | 2.2 ± 0.4 | 2.6 ± 0.4 |
| 2017 | 100^c^ | 4.5 ± 1.7 | | 19.1 ± 3.2 | 1.1 ± 0.2 | 2.7 ± 0.4 | 2.9 ± 0.4 |
|  | 75^c^ | 3.6 ± 1.0 | | 19.2 ± 3.6 | 1.0 ± 0.2 | 2.6 ± 0.5 | 2.8 ± 0.3 |
|  | 50^d^ | 3.6 ± 0.6 | | 18.9 ± 3.0 | 1.0 ± 0.1 | 2.5 ± 0.3 | 2.9 ± 0.3 |
| 2018^e^ | 100 | 3.9 ± 0.7 | | 10.0 ± 1.1 | 1.4 ± 0.1 | 1.0 ± 0.2 | 3.2 ± 0.4 |
|  | 75 | 3.7 ± 0.7 | | 9.8 ± 1.5 | 1.4 ± 0.1 | 1.0 ± 0.2 | 3.0 ± 0.5 |
|  | 50 | 4.1 ± 1.0 | | 10.5 ± 2.2 | 1.7 ± 0.2 | 1.1 ± 0.2 | 3.0 ± 0.6 |
| 2021 Direct seeding | 100 ^f^ | 10.1 ± 2.0 | | 16.1 ± 1.1 | 2.4 ± 0.4 | 2.0 ± 0.2 | 2.7 ± 0.3 |
|  | 75 | 12.2 ± 1.1 | | 17.3 ± 2.6 | 2.9 ± 0.5 | 1.9 ± 0.1 | 2.7 ± 0.3 |
|  | 50 | 14.9 ± 2.2 | | 13.9 ± 1.1 | 3.0 ± 0.3 | 1.5 ± 0.2 | 2.6 ± 0.2 |
| Statistical significance | | | | | | | |
| Y |  | ns | | ns | *** | *** | *** |
| T |  | ns | | ns | * | ns | ns |
| YxT |  | ns | | ns | ** | ns | ns |

Values represent average ^a^(n=12), ^b^(n=9), ^c^(n=18), ^d^(n=16), ^e^(n=18), ^f^(n=4) ± SD.

Table 2S. Concentration of selected micronutrients in shoots of agretti grown for 4 years on saline, B- and Se-laden soil and irrigated with either non-saline water (2016 and 2017) or saline drainage water (2018 and 2021). Statistical difference was performed to analyze the effect of irrigation levels as described in Table 3. ns, *, **, and *** indicate non-significance or significance at 5%, 1%, and 0.1% probability levels, respectively. Y=year, and T= irrigation treatment (%Et_c_).

| Year | Irrigation treatment | Cu | Fe | Mn | Zn |
| --- | --- | --- | --- | --- | --- |
|  | % Et_c_ | ----------------------mg/kg----------------- | | | |
| 2016 | 100^a^ | 10.8 ± 1.4 | 62.6 ± 6.8 | 38.2 ± 7.1 | 18.8 ± 1.6 |
|  | 75^b^ | 11.1 ± 2.2 | 60.8 ± 12.4 | 35.6 ± 7.9 | 19.4 ± 1.8 |
|  | 50^b^ | 11.5 ± 2.0 | 60.8 ± 3.5 | 42.6 ± 7.3 | 19.7 ± 1.2 |
| 2017 | 100^c^ | 10.3 ± 2.4 | 84.2 ± 15.3 | 45.5 ± 8.5 | 20.9 ± 4.1 |
|  | 75^c^ | 9.6 ± 2.5 | 85.3 ± 13.9 | 44.8 ± 8.8 | 21.0 ± 4.0 |
|  | 50^d^ | 10.0 ± 2.5 | 79.9 ± 23.2 | 45.9 ± 7.9 | 20.5 ± 2.9 |
| 2018^e^ | 100 | 11.3 ± 4.0 | 69.3 ± 14.8 | 12.7 ± 2.9 | 14.3 ± 0.8 |
|  | 75 | 11.2 ± 4.0 | 62.4 ± 12.3 | 14.3 ± 4.1 | 15.9 ± 1.4 |
|  | 50 | 10.2 ± 3.2 | 57.8 ± 15.7 | 18.6 ± 6.6 | 17.3 ± 2.4 |
| 2021 Direct seeding | 100 ^f^ | 8.3 ± 1.1 | 195.4 ± 41.2 | 47.6 ± 11.4 | 29.3 ± 2.1 |
|  | 75 | 8.2 ± 0.9 | 228.2 ± 42.7 | 51.6 ± 8.4 | 32.3 ± 0.5 |
|  | 50 | 6.2 ± 0.9 | 131.9 ± 32.5 | 64.0 ± 2.1 | 32.8 ± 2.7 |
| Statistical significance | | | | | |
| Y |  | ** | *** | *** | * |
| T |  | ns | * | ns | ns |
| YxT |  | ns | * | ns | ns |

Values represent average ^a^(n=12), ^b^(n=9), ^c^ (n=18), ^d^(n=16), ^e^(n=18), ^f^(n=4) ± SD.
