# Supplementary figures and images for: Salsola soda as selenium biofortification crop under high saline and boron growing conditions
Source: Front Plant Sci. 2022 Sep 26;13:996502. doi: 10.3389/fpls.2022.996502 (PMC9549694; doi:10.3389/fpls.2022.996502)

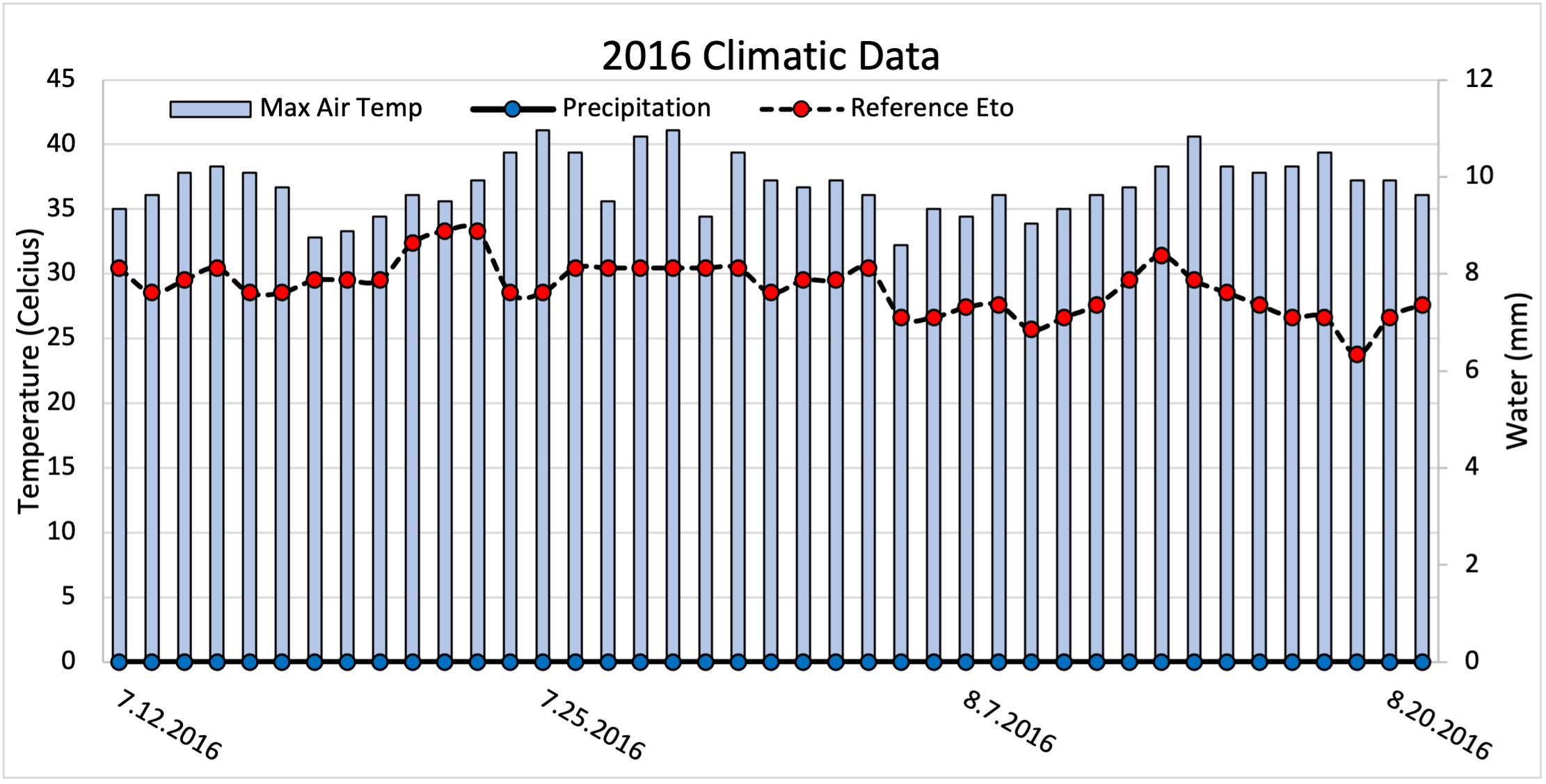

Supplement: Supplementary file 2 [file Data_Sheet_1.zip › SM image 2 2016.jpg]

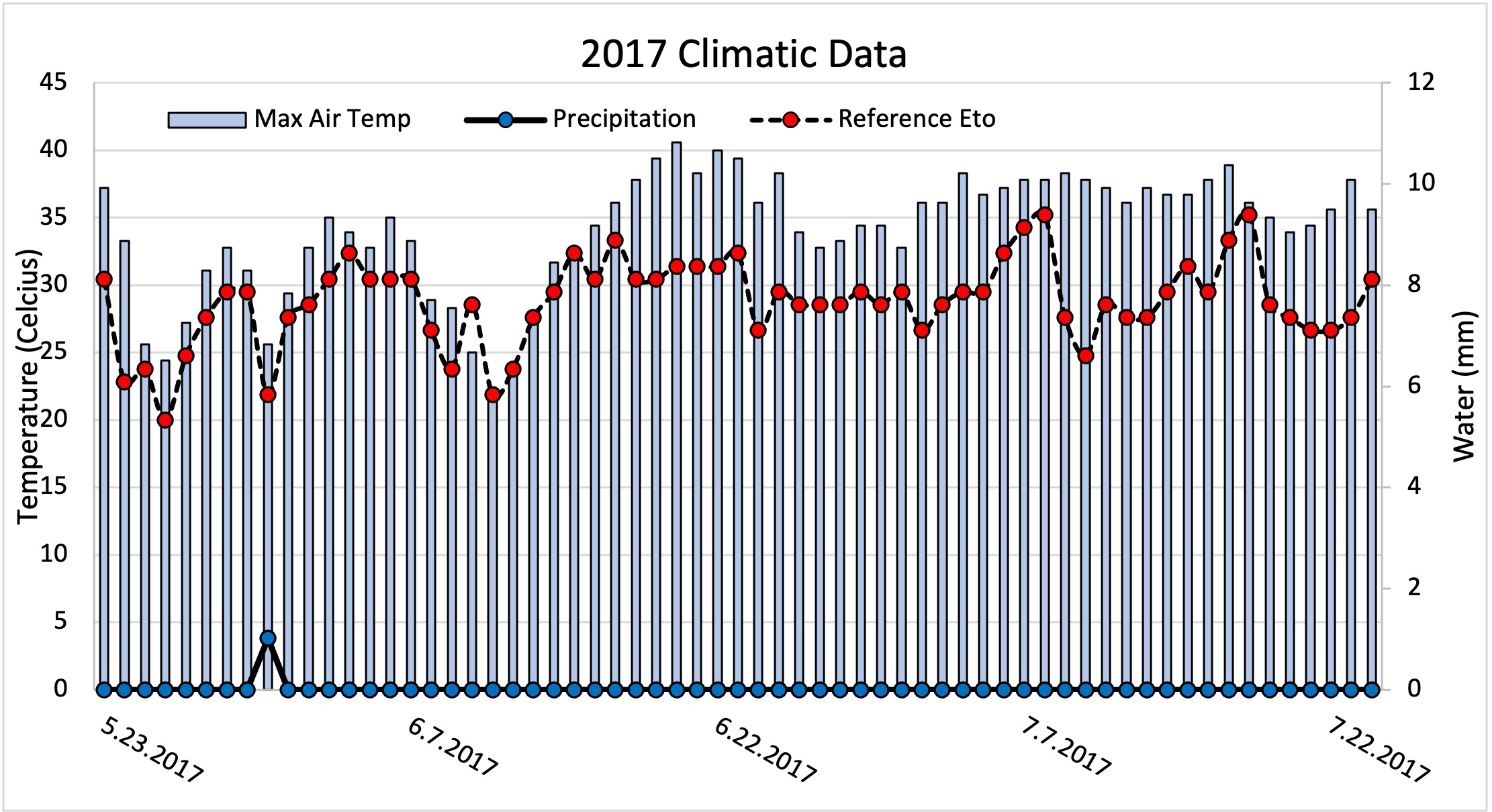

Supplement: Supplementary file 2 [file Data_Sheet_1.zip › SM image 2 2017.jpg]

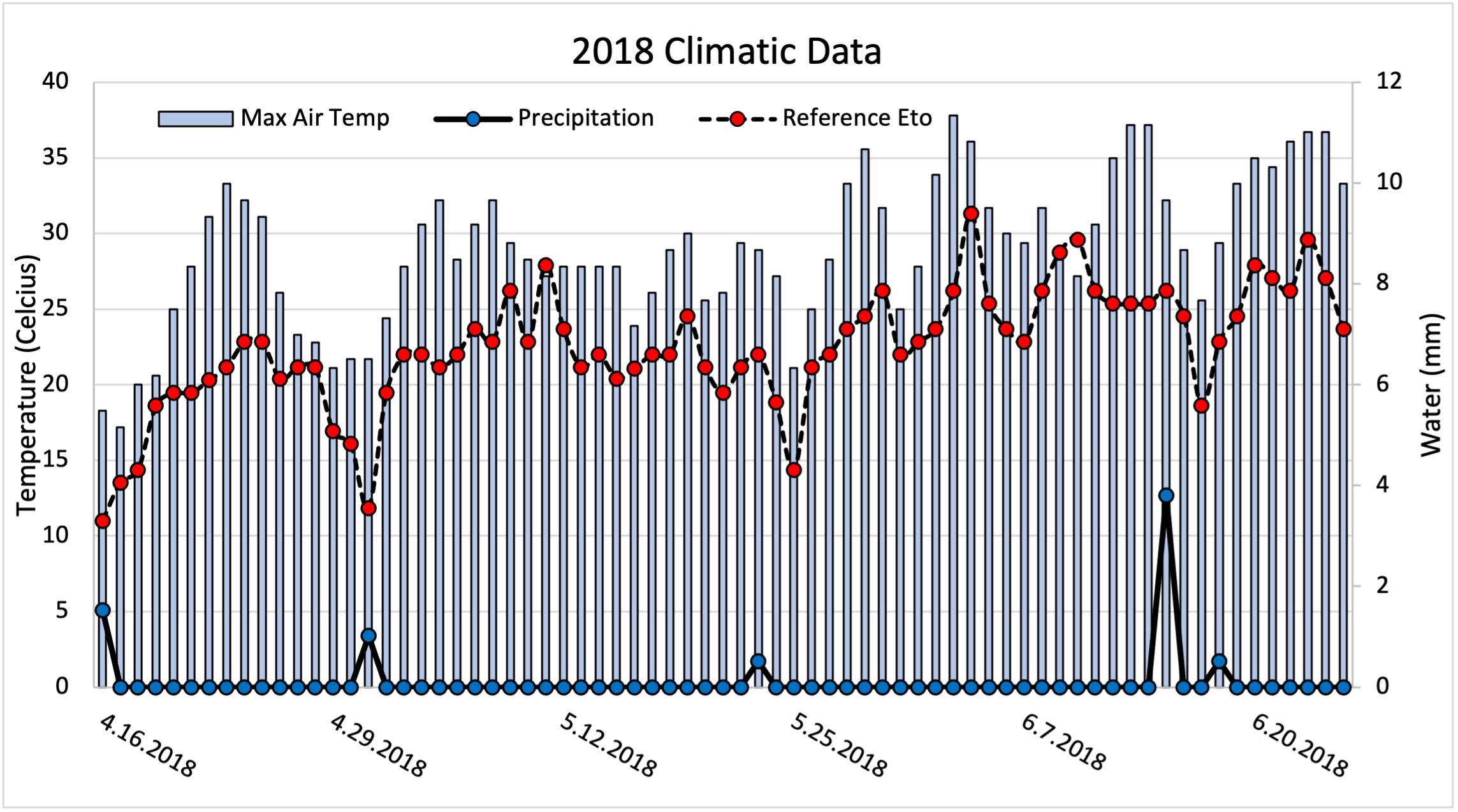

Supplement: Supplementary file 2 [file Data_Sheet_1.zip › SM image 2 2018.jpg]

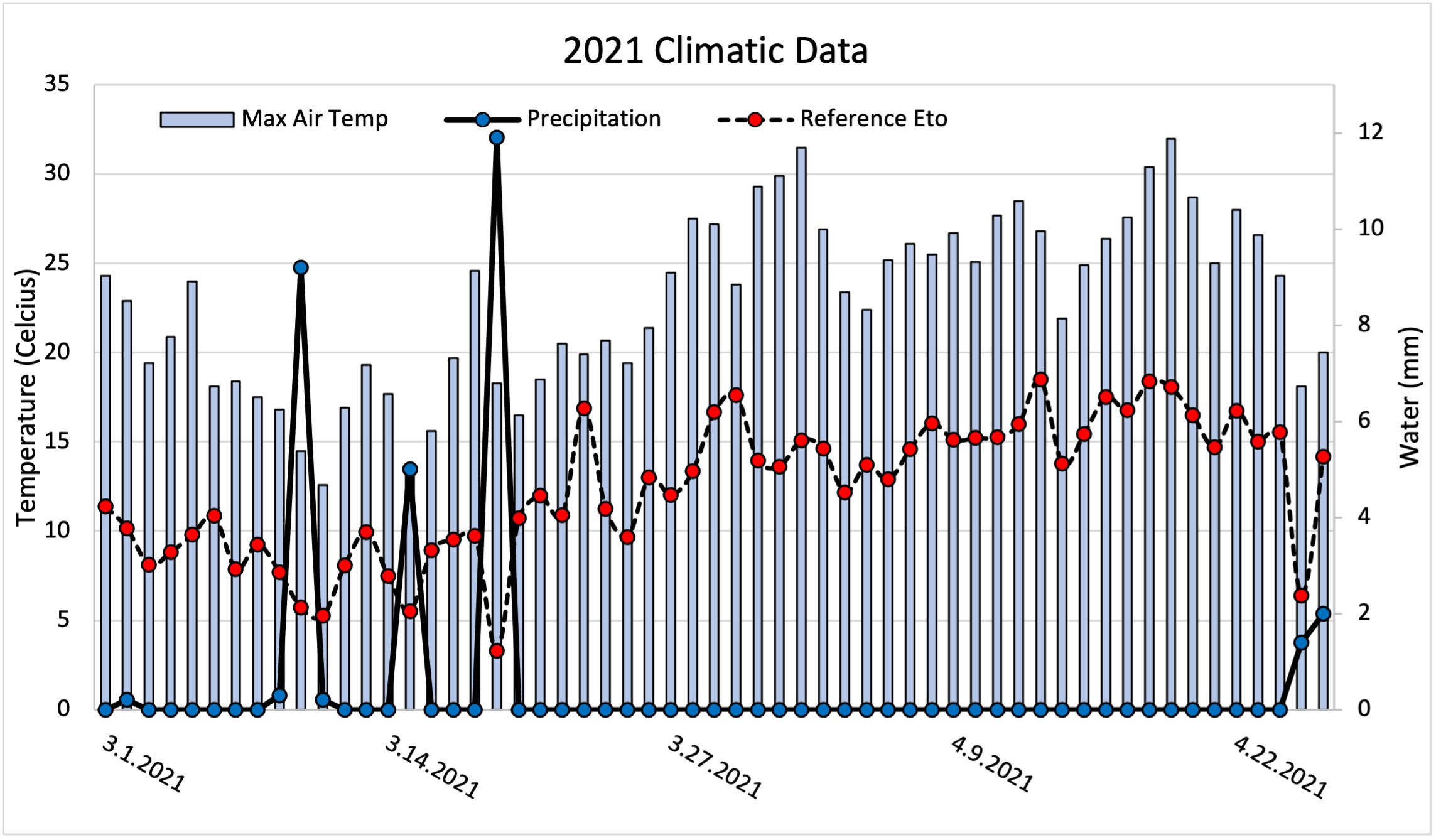

Supplement: Supplementary file 2 [file Data_Sheet_1.zip › SM image 2 2021.jpg]

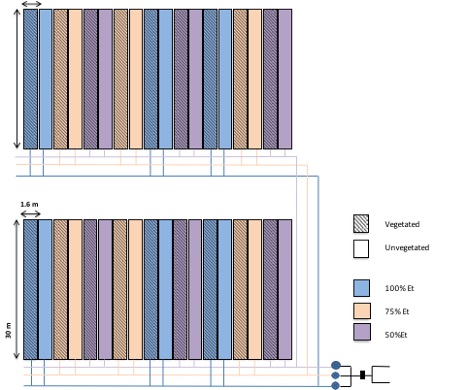

Supplement: Supplementary file 3 [file Image_1.jpg]
